# Commentary: Retinal electrophysiology in central nervous system disorders. A review of human and mouse studies

**DOI:** 10.3389/fnins.2024.1400923

**Published:** 2024-05-07

**Authors:** Charlotte Petit, Marie de Deus, Thomas Schwitzer

**Affiliations:** Pôle Hospitalo-Universitaire de Psychiatrie d'Adulte et d'Addictologie du Grand Nancy, Centre Psychothérapique de Nancy, Laxou, France

**Keywords:** electroretinography, major depressive disorder, treatments, antidepressants, light therapy, retinal function, precision psychiatry

In an interesting review, Constable et al. (2023) recently presented a broad overview of retinal electrophysiology in central nervous system (CNS) disorders, emphasizing the concept of the retina as a window to the brain (London et al., 2013). Indeed, the retina and the brain share a common neuroectodermal origin. Thus, they have similar properties in neurochemistry and neuroanatomy. Authors report variations in latencies, amplitudes of flash electroretinography (fERG), Pattern electroretinography (PERG), multifocal electroretinography (mfERG parameters, and electro-oculography of patients affected by CNS disorders, such as autism, attention deficit hyperactivity disorder, bipolar disorder, schizophrenia, unipolar depression, Parkinson's and Alzheimer's disease. In the review, these variations are also related to genetic modification and exposure to toxic agents in mouse models under these conditions. Changes in retinal chemistry, notably in neurotransmitters, are reviewed in relation to these models. Thanks to these variations, the authors suggest that analyzing ERG signal could contribute to a better understanding of the neurotransmitter anomalies, classification, and evolution of CNS disorders highlighted in the review.

Among these diseases, major depressive disorders (MDD) are relevant to our interests. The authors emphasize the variations in ERG related to MDD treatments, notably antidepressants and light therapy. In this disease, we posit that ERG is probably the key factor in addressing the issue of partial responses to these treatments. This is an extremely crucial matter for discussion and has caught our attention. Through this commentary, we would like to extend this discussion by introducing perspectives that could considerably advance research.

Indeed, MDD are a major public health problem since they affect a significant part of the world's population, estimated at 3.8% (World Health Organization, 2023). Moreover, MDD are one of the most disabling chronic diseases. However, in patients treated with antidepressants or light therapy, the response is frequently unsatisfactory. As a result, changes in treatments and therapeutic strategies are often required for patients to achieve remission. Despite numerous trials, the response remains inadequate for approximately 20% of MDD patients treated for 2 or more years from the onset of the episode (Day et al., 2021). Ineffective therapies tend to prolong the illness. Consequently, MDD patients suffer from persistent depressive symptoms and cognitive dysfunction, leading to an impaired quality of life and a daily functional disability. Additionally, inefficient treatments lead to significant medico-economic costs for society. One of the main challenges in treating MDD patients is monitoring their responses to antidepressants and light therapy. Retinal markers are reliable, reproducible, relevant, easily measurable, and robust. Thus, retinal function measurements could be a solution to this challenge.

In addition, preliminary results exist and have shown different effects of antidepressants on ERG parameters (Moulard et al., 2022). Although promising, these results are insufficient. To complement them, we need to develop ERG biomarkers that could effectively address the issues in monitoring antidepressant responses. To face these crucial challenges of tomorrow, studies must investigate the effects of various classes of antidepressants on ERGs. These studies should be conducted on Selective Serotonin Reuptake Inhibitor (SSRI) and α2 antagonists since they are the most frequently prescribed first-line antidepressants for MDD (Hockenberry et al., 2019; Kazdin et al., 2023). Subjects of these studies should be diagnosed with a current depressive episode needing antidepressant treatments but drug free at the start of the study. The effects of these antidepressants will be assessed by employing ERGs and the standardized Montgomery and Asberg Depression Rating Scale (MADRS), with the findings being correlated. These measurements must be repeated throughout the treatment evaluation period, which lasts 12 weeks for antidepressants. Indeed, the estimated maximum duration of efficacy of an antidepressant is 12 weeks (Kudlow et al., 2014). The strength of this study is the patient being their own control. The findings could provide tools for monitoring the clinical response of antidepressants in unipolar depressed patients. Considering the retina's use of dopamine and serotonin (Wu, 2020), the results may reflect abnormalities in antidepressant-induced neurotransmission. The retina also uses neurosteroids (Bucolo and Drago, 2004) which are normalized with the use of SSRIs (Van Broekhoven and Verkes, 2003). Thus, potential ERG variations observed with antidepressants could be explained by their impact on neurosteroids.

Light therapy is another first-line reference treatment in MDD (Lam et al., 2016). Its effectiveness is based on its action on melatonin and the circadian rhythm. Interestingly, the retina uses melatonin to detect daylight (Wu, 2020). Thus, melatoninergic modifications provoked by light therapy could also cause ERG variations. Markers of response to light therapy can therefore be found in ERGs. To address these crucial issues, clinical studies that include ERG measurements need to be carried out on MDD patients treated with light therapy. The participants in these studies should be individuals with non-seasonal MDD who have not previously been treated with light therapy. As part of their standard care, patients will be treated with daily light therapy or a placebo device in a double-blind study to assess the real effect of active light therapy. For this purpose, ERG correlated with a standardized MADRS will be assessed on MDD patients during different weeks. These measurements must be repeated up to 8 weeks in non-seasonal MDD patients to determine the efficacy of light therapy (Lam et al., 2016). These data could provide ERG markers to monitor light therapy efficacy over time in non-seasonal MDD patients. Indeed, melatonin is produced in response to the retina detecting daylight. Light therapy also acts on the melatoninergic pathway, Thus, ERG results may reflect light therapy-induced melatoninergic modifications.

As mentioned above, monoaminergic, neurosteroidergic, and melatoninergic modifications impact retina function. Evaluating its functioning could therefore provide markers of the therapeutic response to antidepressants and light therapy in MDD patients. Indeed, ERG profiles could be used to categorize subgroups of patients as responders and non-responders to antidepressant types and/or light therapy. This could help clinicians assess the potential response of MDD patients to these treatments, knowing if and when to change the treatment. Thus, the therapeutic strategy in the follow-up would be adapted more rapidly. This may allow better-targeted therapeutic interventions for the benefit of patients and the clinicians. Thus, ERG measures could lead toward precision psychiatry, thanks to their robustness, reproducibility, and reliability (Schwitzer et al., 2022).

## Author contributions

CP: Conceptualization, Formal analysis, Writing – original draft. MD: Conceptualization, Formal analysis, Writing – original draft. TS: Conceptualization, Supervision, Validation, Writing – review & editing.
